# [μ-Bis(diphenyl­arsino)methane-1:2κ^2^ *As*:*As*′]nona­carbonyl-1κ^3^ *C*,2κ^3^ *C*,3κ^3^ *C*-[tris­(4-fluoro­phen­yl)phosphine-3κ*P*]-*triangulo*-triruthenium(0)

**DOI:** 10.1107/S1600536809047229

**Published:** 2009-11-21

**Authors:** Omar bin Shawkataly, Imthyaz Ahmed Khan, Chin Sing Yeap, Hoong-Kun Fun

**Affiliations:** a Chemical Sciences Programme, School of Distance Education, Universiti Sains Malaysia, 11800 USM, Penang, Malaysia; b X-ray Crystallography Unit, School of Physics, Universiti Sains Malaysia, 11800 USM, Penang, Malaysia

## Abstract

In the title *triangulo*-triruthenium compound, [Ru_3_(C_25_H_22_(As_2_)(C_18_H_12_F_3_P)(CO)_9_], the bis­(diphenyl­arsino)methane ligand bridges an Ru—Ru bond and the monodentate phosphine ligand bonds to the third Ru atom. Both the phosphine and arsine ligands are equatorial with respect to the Ru_3_ triangle. Additionally, each Ru atom carries one equatorial and two axial terminal carbonyl ligands. The three phosphine-substituted rings make dihedral angles of 87.76 (13), 57.43 (13) and 73.81 (12)° with each other. The dihedral angles between the pairs of rings are 69.78 (14) and 83.38 (16)° for the two diphenyl­arsino groups. In the crystal packing, mol­ecules are linked by inter­molecular C—H⋯F and C—H⋯O hydrogen bonds, forming two-dimensional planes parallel to the *ab* plane. These planes are also linked by inter­molecular C—H⋯O hydrogen bonds into a three-dimensional framework. Inter­molecular C—H⋯π inter­actions further stabilize the crystal structure.

## Related literature

For general background to *triangulo*-triruthenium derivatives, see: Bruce (1985 ▶, 1988*a* ▶,*b* ▶); Shawkataly *et al.* (1998 ▶). For related structures, see: Shawkataly *et al.* (2006 ▶, 2009*a* ▶,*b* ▶). For the synthesis of bis­(diphenyl­arsino)methane, see: Bruce *et al.* (1983 ▶). For details of the Cambridge Structural Database, see: Allen (2002 ▶).
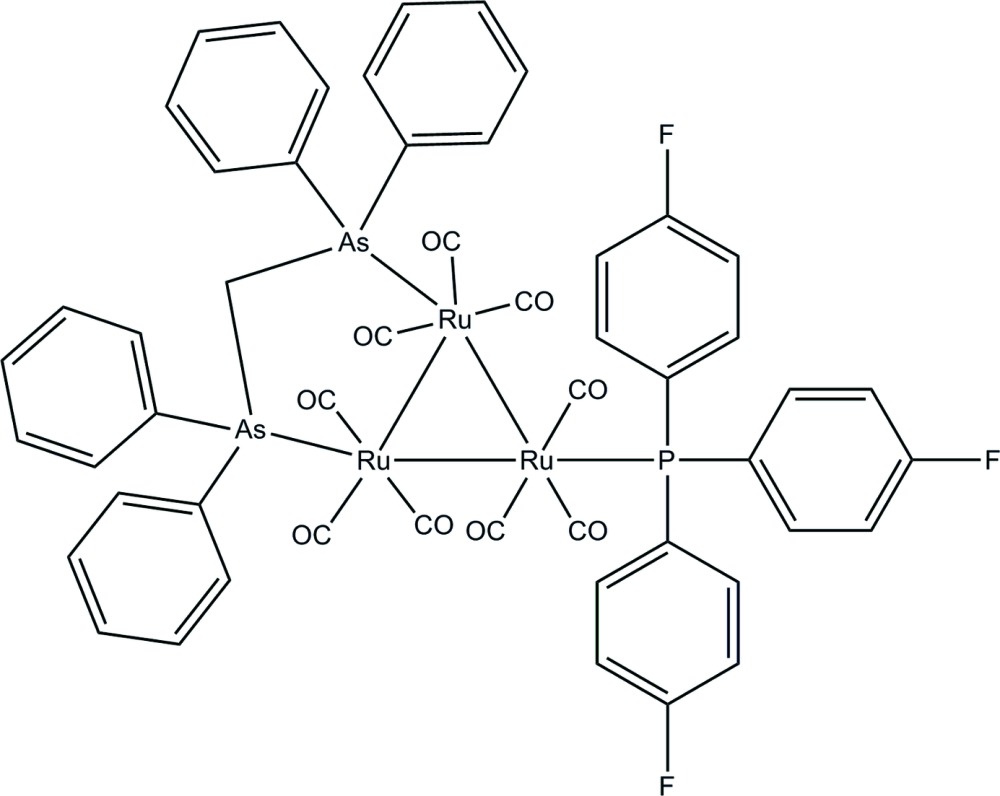


## Experimental

### 

#### Crystal data

[Ru_3_(C_25_H_22_As_2_)(C_18_H_12_F_3_P)(CO)_9_]
*M* *_r_* = 1343.81
Monoclinic, 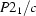

*a* = 16.3071 (2) Å
*b* = 16.8142 (2) Å
*c* = 18.8085 (2) Å
β = 98.105 (1)°
*V* = 5105.6 (1) Å^3^
*Z* = 4
Mo *K*α radiation
μ = 2.26 mm^−1^
*T* = 296 K
0.38 × 0.25 × 0.14 mm

#### Data collection

Bruker SMART APEXII CCD area-detector diffractometer
Absorption correction: multi-scan (**SADABS**; Bruker, 2005 ▶) *T*_min_ = 0.478, *T*_max_ = 0.740
89126 measured reflections
20637 independent reflections
13049 reflections with *I* > 2σ(*I*)
*R*_int_ = 0.032

#### Refinement

*R*[*F* ^2^ > 2σ(*F* ^2^)] = 0.032
*wR*(*F* ^2^) = 0.067
*S* = 1.00
20637 reflections
631 parameters
H-atom parameters constrained
Δρ_max_ = 0.44 e Å^−3^
Δρ_min_ = −0.54 e Å^−3^

### 

Data collection: *APEX2* (Bruker, 2005 ▶); cell refinement: *SAINT* (Bruker, 2005 ▶); data reduction: *SAINT*; program(s) used to solve structure: *SHELXTL* (Sheldrick, 2008 ▶); program(s) used to refine structure: *SHELXTL*; molecular graphics: *SHELXTL* software used to prepare material for publication: *SHELXTL* and *PLATON* (Spek, 2009 ▶).

## Supplementary Material

Crystal structure: contains datablocks global, I. DOI: 10.1107/S1600536809047229/sj2667sup1.cif

Structure factors: contains datablocks I. DOI: 10.1107/S1600536809047229/sj2667Isup2.hkl

Additional supplementary materials:  crystallographic information; 3D view; checkCIF report

## Figures and Tables

**Table 1:** Hydrogen-bond geometry (Å, °)

*D*—H⋯*A*	*D*—H	H⋯*A*	*D*⋯*A*	*D*—H⋯*A*
C23—H23*A*⋯F3^i^	0.93	2.46	3.337 (5)	157
C34—H34*A*⋯O3^ii^	0.93	2.58	3.405 (3)	148
C42—H42*A*⋯O2^iii^	0.93	2.52	3.438 (3)	167
C11—H11*A*⋯*Cg*1^iv^	0.93	2.94	3.767 (3)	149
C28—H28*A*⋯*Cg*2^ii^	0.93	2.89	3.807 (3)	168

Symmetry codes: (i) 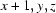; (ii) 
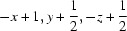
; (iii) 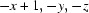; (iv) 
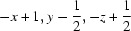
. *Cg*1 and *Cg*2 are the centroids of the C38–C43 and C1–C6 phenyl rings, respectively.

**Fig. 1.**
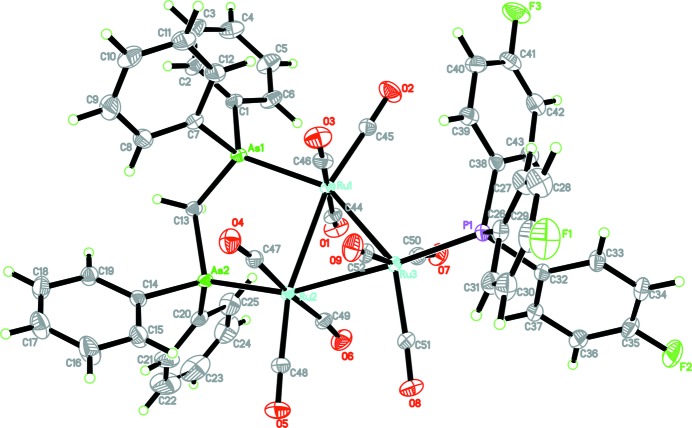
The molecular structure of the title compound with 20% probability ellipsoids for non-H atoms.

**Fig. 2.**
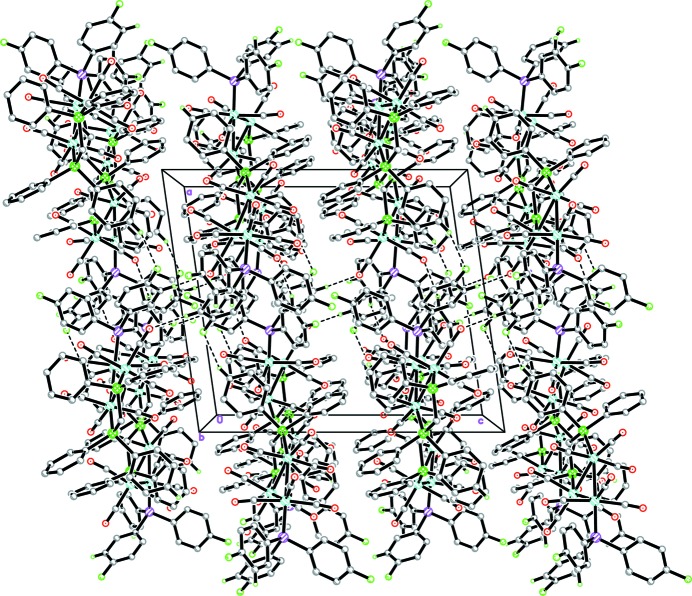
The crystal packing of the title compound, viewed down the b axis, showing the molecules packed into 3-D framework. Hydrogen atoms not involved in the hydrogen-bonding (dashed lines) have been omitted for clarity.

## supplementary crystallographic information

### Comment

Tri-angulotriruthenium clusters are known for their interesting structural variations and related catalytic activity. A large number of substituted derivatives, Ru_3_(CO)_12-n_*L*_n_ (*L*=15 group Ligand) have been reported (Bruce *et al.*, 1988*a*,b; 1985). In continuation of our interest in the substituted clusters (Shawkataly *et al.*, 1998) we report the synthesis and structure of Ru_3_(CO)_9_(µ-Ph_2_AsCH_2_AsPh_2_)(P(4-FC_6_H_4_)_3_). The Cambridge Structural Database (Allen, 2002) revealed no structure of the above cluster.

The bond lengths and angles of title compound (Fig. 1) are comparable to its related structures (Shawkataly *et al.*, 2006; Shawkataly *et al.*, 2009*a*,b). The bis(diphenylarsino)methane ligand bridges the Ru1—Ru2 bond and the monodentate phosphine ligand bonds to the Ru3 atom. Both the phosphine and arsine ligands are equatorial with respect to the Ru_3_ triangle. Additionally, each Ru atom carries one equatorial and two axial terminal carbonyl ligands The three phosphine substituted phenyl rings (C26–C31, C32–C37 and C38–C43) make dihedral angles (C26–C31/C32–C37, C26–C31/C38–C43 and C32–C37/C38–C43) of 87.76 (13), 57.43 (13) and 73.81 (12)° with each other respectively. The dihedral angles between the two phenyl rings (C1–C6/C7–C12 and C14–C19/C20–C25) are 69.78 (14) and 83.38 (16)° for the two diphenylarsino groups respectively.

In the crystal packing (Fig. 2), the molecules are linked by intermolecular C23—H23A···F3 and C34—H34A···O3 hydrogen bonds to form two-dimensional planes parallel to *ab* plane. These planes are further linked by intermolecular C42—H42A···O2 hydrogen bonds into a three-dimensional framework. Intermolecular C—H···π interactions further stabilize the crystal structure (Table 1).

### Experimental

Tris(4-flurophenyl)phosphine (Strem chemicals) is used as received and bis(diphenylarsino)methane (Bruce *et al.*, 1983) was prepared by reported procedure. Ru_3_(CO)_10_(µ-Ph_2_AsCH_2_AsPh_2_) (105.5 mg, 0.1 mmol) and tris(4-flurophenyl)phosphine (31.62 mg, 0.1 mmol) were refluxed for 15 minutes in hexane (25 ml) under a current of nitrogen. The reaction mixture turned intense red. The solvent was removed under vacuum. The reaction mixture was separated by TLC (dichloromethane:hexane, 30:70). Two bands appeared. The major band (red) *R*_f_ = 0.56 yielded the title compound which was crystallized from CH_2_Cl_2_—CH_3_OH, yield = 58 mg, 42.29%, m.p. 190 °C. IR(cyclohexane).ν(CO) 2054 s, 1993 s, 1976 s cm^-1^. 1H NMR (CDCl_3_), δ 7.32–7.50 (m, 26H, Ph), δ 7.08–7.13 (t, 6H, Ph), δ 4.01 (s, 2H, –CH_2_–).

### Refinement

All hydrogen atoms were positioned geometrically and refined using a riding model with C—H = 0.93–0.97 Å and *U*_iso_(H) = 1.2 *U*_eq_(C).

### Crystal data

**Table:**

[Ru_3_(C_25_H_22_As_2_)(C_18_H_12_F_3_P)(CO)_9_]	*F*(000) = 2632
*M**_r_* = 1343.81	*D*_x_ = 1.748 Mg m^−^^3^
Monoclinic, *P*2_1_/*c*	Mo *K*α radiation, λ = 0.71073 Å
Hall symbol: -P 2ybc	Cell parameters from 9910 reflections
*a* = 16.3071 (2) Å	θ = 2.5–31.1°
*b* = 16.8142 (2) Å	µ = 2.26 mm^−^^1^
*c* = 18.8085 (2) Å	*T* = 296 K
β = 98.105 (1)°	Plate, red
*V* = 5105.6 (1) Å^3^	0.38 × 0.25 × 0.14 mm
*Z* = 4	

### Data collection

**Table:**

Bruker SMART APEXII CCD area-detector diffractometer	20637 independent reflections
Radiation source: fine-focus sealed tube	13049 reflections with *I* > 2σ(*I*)
graphite	*R*_int_ = 0.032
φ and ω scans	θ_max_ = 34.0°, θ_min_ = 1.6°
Absorption correction: multi-scan (*SADABS*; Bruker, 2005)	*h* = −25→25
*T*_min_ = 0.478, *T*_max_ = 0.740	*k* = −23→26
89126 measured reflections	*l* = −29→22

### Refinement

**Table:**

Refinement on *F*^2^	Primary atom site location: structure-invariant direct methods
Least-squares matrix: full	Secondary atom site location: difference Fourier map
*R*[*F*^2^ > 2σ(*F*^2^)] = 0.032	Hydrogen site location: inferred from neighbouring sites
*wR*(*F*^2^) = 0.067	H-atom parameters constrained
*S* = 1.00	*w* = 1/[σ^2^(*F*_o_^2^) + (0.0231*P*)^2^ + 1.7596*P*] where *P* = (*F*_o_^2^ + 2*F*_c_^2^)/3
20637 reflections	(Δ/σ)_max_ = 0.002
631 parameters	Δρ_max_ = 0.44 e Å^−^^3^
0 restraints	Δρ_min_ = −0.54 e Å^−^^3^

### Special details

**Table:**

Geometry. All e.s.d.'s (except the e.s.d. in the dihedral angle between two l.s. planes) are estimated using the full covariance matrix. The cell e.s.d.'s are taken into account individually in the estimation of e.s.d.'s in distances, angles and torsion angles; correlations between e.s.d.'s in cell parameters are only used when they are defined by crystal symmetry. An approximate (isotropic) treatment of cell e.s.d.'s is used for estimating e.s.d.'s involving l.s. planes.
Refinement. Refinement of *F*^2^ against ALL reflections. The weighted *R*-factor *wR* and goodness of fit *S* are based on *F*^2^, conventional *R*-factors *R* are based on *F*, with *F* set to zero for negative *F*^2^. The threshold expression of *F*^2^ > σ(*F*^2^) is used only for calculating *R*-factors(gt) *etc*. and is not relevant to the choice of reflections for refinement. *R*-factors based on *F*^2^ are statistically about twice as large as those based on *F*, and *R*- factors based on ALL data will be even larger.

### Fractional atomic coordinates and isotropic or equivalent isotropic displacement parameters (Å^2^)

**Table:**

	*x*	*y*	*z*	*U*_iso_*/*U*_eq_	
Ru1	0.758329 (9)	−0.076419 (9)	0.204485 (9)	0.03449 (4)	
Ru2	0.901268 (9)	0.000219 (9)	0.278744 (9)	0.03474 (4)	
Ru3	0.749043 (9)	0.084526 (9)	0.254257 (9)	0.03606 (4)	
As1	0.822101 (12)	−0.206958 (11)	0.223901 (11)	0.03432 (5)	
As2	0.996032 (11)	−0.110301 (12)	0.269926 (12)	0.03624 (5)	
P1	0.61915 (3)	0.14500 (3)	0.23231 (3)	0.03820 (12)	
F1	0.47921 (13)	0.24586 (13)	0.49280 (9)	0.1049 (7)	
F2	0.58974 (12)	0.44950 (10)	0.07022 (12)	0.1059 (7)	
F3	0.38045 (10)	−0.06529 (11)	0.05565 (11)	0.0960 (6)	
O1	0.84836 (12)	−0.05511 (11)	0.07344 (10)	0.0695 (5)	
O2	0.60490 (12)	−0.12226 (13)	0.10357 (11)	0.0806 (6)	
O3	0.66519 (12)	−0.10095 (12)	0.33318 (11)	0.0714 (5)	
O4	0.85047 (11)	−0.08172 (11)	0.41116 (10)	0.0681 (5)	
O5	1.00783 (13)	0.11974 (12)	0.36958 (12)	0.0836 (6)	
O6	0.95116 (12)	0.08300 (11)	0.14619 (11)	0.0703 (5)	
O7	0.74552 (12)	0.09629 (11)	0.09135 (10)	0.0662 (5)	
O8	0.85238 (12)	0.22948 (10)	0.29874 (11)	0.0750 (6)	
O9	0.71744 (12)	0.05435 (11)	0.40925 (10)	0.0693 (5)	
C1	0.79099 (13)	−0.28842 (12)	0.15128 (12)	0.0415 (5)	
C2	0.79025 (18)	−0.36784 (14)	0.16849 (15)	0.0647 (7)	
H2A	0.8061	−0.3839	0.2157	0.078*	
C3	0.7660 (2)	−0.42404 (16)	0.11581 (18)	0.0807 (9)	
H3A	0.7646	−0.4776	0.1280	0.097*	
C4	0.74428 (19)	−0.40124 (17)	0.04610 (17)	0.0736 (8)	
H4A	0.7286	−0.4392	0.0108	0.088*	
C5	0.7455 (2)	−0.32306 (17)	0.02835 (15)	0.0733 (8)	
H5A	0.7308	−0.3074	−0.0192	0.088*	
C6	0.76868 (17)	−0.26642 (15)	0.08092 (13)	0.0589 (6)	
H6A	0.7691	−0.2129	0.0684	0.071*	
C7	0.80087 (12)	−0.26512 (12)	0.30795 (11)	0.0397 (4)	
C8	0.86048 (15)	−0.29136 (15)	0.36136 (13)	0.0580 (6)	
H8A	0.9156	−0.2775	0.3608	0.070*	
C9	0.8391 (2)	−0.33831 (19)	0.41617 (15)	0.0769 (8)	
H9A	0.8799	−0.3560	0.4522	0.092*	
C10	0.7580 (2)	−0.35891 (17)	0.41770 (15)	0.0720 (8)	
H10A	0.7441	−0.3915	0.4541	0.086*	
C11	0.69801 (18)	−0.33164 (18)	0.36595 (17)	0.0733 (8)	
H11A	0.6429	−0.3446	0.3675	0.088*	
C12	0.71888 (15)	−0.28477 (16)	0.31108 (15)	0.0626 (7)	
H12A	0.6776	−0.2662	0.2758	0.075*	
C13	0.94271 (12)	−0.20738 (12)	0.22724 (12)	0.0398 (4)	
H13A	0.9559	−0.2133	0.1788	0.048*	
H13B	0.9653	−0.2530	0.2549	0.048*	
C14	1.05731 (12)	−0.14627 (13)	0.36072 (12)	0.0435 (5)	
C15	1.06977 (17)	−0.09360 (17)	0.41720 (16)	0.0715 (8)	
H15A	1.0507	−0.0416	0.4108	0.086*	
C16	1.1103 (2)	−0.1172 (2)	0.48308 (17)	0.0928 (11)	
H16A	1.1191	−0.0808	0.5206	0.111*	
C17	1.13761 (19)	−0.1938 (2)	0.49360 (16)	0.0802 (9)	
H17A	1.1637	−0.2100	0.5385	0.096*	
C18	1.12629 (17)	−0.24650 (17)	0.43766 (15)	0.0682 (7)	
H18A	1.1451	−0.2985	0.4444	0.082*	
C19	1.08719 (14)	−0.22290 (14)	0.37142 (13)	0.0516 (6)	
H19A	1.0808	−0.2588	0.3335	0.062*	
C20	1.08183 (13)	−0.09446 (13)	0.20931 (15)	0.0499 (6)	
C21	1.16319 (16)	−0.08505 (17)	0.2382 (2)	0.0799 (9)	
H21A	1.1784	−0.0872	0.2877	0.096*	
C22	1.2224 (2)	−0.0724 (3)	0.1941 (3)	0.1253 (17)	
H22A	1.2776	−0.0664	0.2140	0.150*	
C23	1.2010 (3)	−0.0686 (3)	0.1217 (3)	0.130 (2)	
H23A	1.2417	−0.0599	0.0925	0.157*	
C24	1.1203 (3)	−0.0775 (2)	0.0913 (2)	0.1048 (13)	
H24A	1.1057	−0.0752	0.0417	0.126*	
C25	1.06032 (19)	−0.09009 (18)	0.13602 (18)	0.0756 (9)	
H25A	1.0051	−0.0956	0.1161	0.091*	
C26	0.57217 (14)	0.17311 (13)	0.31183 (12)	0.0466 (5)	
C27	0.49231 (16)	0.15401 (17)	0.32221 (14)	0.0632 (7)	
H27A	0.4591	0.1246	0.2875	0.076*	
C28	0.46076 (19)	0.1780 (2)	0.38355 (16)	0.0776 (9)	
H28A	0.4073	0.1642	0.3907	0.093*	
C29	0.5098 (2)	0.22193 (18)	0.43266 (14)	0.0721 (8)	
C30	0.5882 (2)	0.24341 (17)	0.42481 (15)	0.0709 (8)	
H30A	0.6200	0.2740	0.4596	0.085*	
C31	0.61979 (17)	0.21875 (15)	0.36371 (14)	0.0597 (6)	
H31A	0.6734	0.2329	0.3574	0.072*	
C32	0.61221 (12)	0.24017 (12)	0.18371 (12)	0.0426 (5)	
C33	0.53927 (15)	0.28460 (14)	0.17773 (14)	0.0552 (6)	
H33A	0.4951	0.2664	0.1995	0.066*	
C34	0.53190 (17)	0.35528 (15)	0.13977 (15)	0.0649 (7)	
H34A	0.4834	0.3850	0.1361	0.078*	
C35	0.59754 (18)	0.38056 (15)	0.10780 (16)	0.0664 (7)	
C36	0.66977 (16)	0.33947 (15)	0.11234 (15)	0.0636 (7)	
H36A	0.7135	0.3583	0.0903	0.076*	
C37	0.67676 (14)	0.26870 (14)	0.15073 (13)	0.0526 (6)	
H37A	0.7259	0.2399	0.1543	0.063*	
C38	0.54110 (12)	0.08408 (13)	0.17909 (12)	0.0406 (5)	
C39	0.51251 (14)	0.01470 (15)	0.20801 (14)	0.0541 (6)	
H39A	0.5307	0.0019	0.2557	0.065*	
C40	0.45758 (15)	−0.03552 (16)	0.16702 (17)	0.0644 (7)	
H40A	0.4379	−0.0813	0.1866	0.077*	
C41	0.43315 (14)	−0.01542 (17)	0.09671 (16)	0.0621 (7)	
C42	0.46057 (15)	0.05001 (17)	0.06592 (14)	0.0579 (6)	
H42A	0.4432	0.0610	0.0177	0.069*	
C43	0.51485 (13)	0.10050 (14)	0.10721 (12)	0.0472 (5)	
H43A	0.5339	0.1459	0.0866	0.057*	
C44	0.81765 (14)	−0.05964 (13)	0.12443 (13)	0.0472 (5)	
C45	0.66118 (14)	−0.10384 (14)	0.14291 (13)	0.0491 (5)	
C46	0.70225 (14)	−0.08582 (14)	0.28761 (14)	0.0497 (5)	
C47	0.86518 (12)	−0.05113 (13)	0.36024 (13)	0.0444 (5)	
C48	0.96867 (14)	0.07385 (14)	0.33563 (14)	0.0523 (6)	
C49	0.92893 (13)	0.05136 (13)	0.19364 (14)	0.0479 (5)	
C50	0.75062 (14)	0.08463 (13)	0.15216 (14)	0.0479 (5)	
C51	0.81181 (14)	0.17562 (13)	0.28089 (13)	0.0471 (5)	
C52	0.73155 (14)	0.06203 (13)	0.35212 (14)	0.0476 (5)	

### Atomic displacement parameters (Å^2^)

**Table:**

	*U*^11^	*U*^22^	*U*^33^	*U*^12^	*U*^13^	*U*^23^
Ru1	0.03469 (7)	0.02893 (8)	0.03910 (9)	−0.00302 (6)	0.00266 (6)	−0.00330 (7)
Ru2	0.03017 (7)	0.02739 (8)	0.04667 (10)	−0.00148 (5)	0.00549 (6)	−0.00464 (7)
Ru3	0.03345 (7)	0.02839 (8)	0.04538 (10)	0.00152 (6)	0.00223 (7)	−0.00243 (7)
As1	0.03681 (9)	0.02778 (10)	0.03869 (11)	−0.00436 (7)	0.00647 (8)	−0.00183 (8)
As2	0.03112 (9)	0.02945 (10)	0.04899 (13)	−0.00058 (7)	0.00856 (9)	−0.00339 (9)
P1	0.0356 (2)	0.0364 (3)	0.0421 (3)	0.0036 (2)	0.0036 (2)	−0.0004 (2)
F1	0.1420 (17)	0.1256 (16)	0.0524 (10)	0.0436 (14)	0.0324 (11)	−0.0097 (10)
F2	0.1104 (14)	0.0618 (11)	0.1438 (18)	0.0148 (10)	0.0117 (13)	0.0494 (11)
F3	0.0637 (10)	0.1025 (14)	0.1172 (15)	−0.0194 (9)	−0.0035 (10)	−0.0525 (12)
O1	0.0940 (14)	0.0663 (12)	0.0538 (11)	−0.0166 (10)	0.0300 (10)	−0.0113 (9)
O2	0.0611 (11)	0.0898 (15)	0.0816 (14)	−0.0028 (10)	−0.0220 (10)	−0.0238 (12)
O3	0.0743 (12)	0.0746 (13)	0.0734 (13)	−0.0144 (10)	0.0382 (11)	−0.0073 (10)
O4	0.0722 (12)	0.0739 (13)	0.0611 (12)	0.0065 (9)	0.0192 (10)	0.0154 (10)
O5	0.0797 (13)	0.0609 (12)	0.1027 (16)	−0.0200 (10)	−0.0135 (12)	−0.0250 (11)
O6	0.0775 (12)	0.0568 (11)	0.0818 (14)	−0.0046 (9)	0.0287 (11)	0.0182 (10)
O7	0.0889 (13)	0.0570 (11)	0.0523 (11)	0.0062 (9)	0.0091 (10)	0.0036 (9)
O8	0.0824 (13)	0.0430 (10)	0.0953 (15)	−0.0199 (9)	−0.0021 (11)	−0.0059 (10)
O9	0.0901 (14)	0.0657 (12)	0.0529 (11)	0.0191 (10)	0.0123 (10)	0.0093 (9)
C1	0.0460 (11)	0.0319 (11)	0.0477 (13)	−0.0054 (8)	0.0105 (9)	−0.0093 (9)
C2	0.095 (2)	0.0357 (13)	0.0606 (16)	−0.0064 (12)	−0.0008 (14)	−0.0063 (12)
C3	0.120 (3)	0.0358 (15)	0.085 (2)	−0.0107 (15)	0.008 (2)	−0.0127 (14)
C4	0.096 (2)	0.0546 (17)	0.073 (2)	−0.0207 (15)	0.0233 (17)	−0.0319 (15)
C5	0.115 (2)	0.0596 (18)	0.0469 (15)	−0.0188 (16)	0.0186 (16)	−0.0166 (13)
C6	0.0906 (18)	0.0412 (13)	0.0475 (14)	−0.0137 (12)	0.0185 (13)	−0.0087 (11)
C7	0.0465 (11)	0.0316 (10)	0.0421 (12)	−0.0043 (8)	0.0104 (9)	0.0004 (9)
C8	0.0531 (13)	0.0669 (17)	0.0532 (15)	−0.0070 (12)	0.0051 (12)	0.0111 (13)
C9	0.084 (2)	0.089 (2)	0.0557 (17)	0.0009 (17)	0.0038 (15)	0.0246 (16)
C10	0.098 (2)	0.0651 (19)	0.0574 (17)	−0.0151 (16)	0.0267 (16)	0.0148 (14)
C11	0.0660 (16)	0.076 (2)	0.081 (2)	−0.0195 (14)	0.0237 (15)	0.0220 (17)
C12	0.0501 (13)	0.0680 (17)	0.0704 (17)	−0.0111 (12)	0.0108 (12)	0.0202 (14)
C13	0.0379 (9)	0.0331 (11)	0.0496 (12)	−0.0014 (8)	0.0105 (9)	−0.0053 (9)
C14	0.0369 (10)	0.0432 (12)	0.0496 (13)	0.0025 (8)	0.0032 (9)	−0.0054 (10)
C15	0.0713 (17)	0.0582 (17)	0.076 (2)	0.0199 (13)	−0.0196 (15)	−0.0224 (14)
C16	0.096 (2)	0.097 (2)	0.073 (2)	0.0368 (19)	−0.0300 (18)	−0.0384 (19)
C17	0.083 (2)	0.095 (2)	0.0556 (17)	0.0251 (17)	−0.0122 (15)	−0.0056 (17)
C18	0.0735 (17)	0.0595 (17)	0.0684 (19)	0.0149 (13)	−0.0006 (14)	0.0081 (14)
C19	0.0577 (13)	0.0448 (13)	0.0512 (14)	0.0078 (10)	0.0036 (11)	−0.0050 (11)
C20	0.0449 (11)	0.0327 (11)	0.0769 (18)	0.0009 (9)	0.0252 (12)	0.0005 (11)
C21	0.0491 (14)	0.077 (2)	0.118 (3)	−0.0181 (13)	0.0283 (16)	−0.0133 (18)
C22	0.065 (2)	0.124 (4)	0.200 (5)	−0.026 (2)	0.065 (3)	−0.012 (4)
C23	0.108 (3)	0.101 (3)	0.209 (6)	−0.006 (2)	0.115 (4)	0.019 (4)
C24	0.123 (3)	0.096 (3)	0.113 (3)	0.016 (2)	0.080 (3)	0.024 (2)
C25	0.0691 (17)	0.082 (2)	0.084 (2)	0.0072 (15)	0.0394 (16)	0.0145 (17)
C26	0.0513 (12)	0.0468 (13)	0.0409 (12)	0.0136 (10)	0.0040 (10)	0.0005 (10)
C27	0.0565 (14)	0.085 (2)	0.0494 (15)	0.0059 (13)	0.0112 (12)	−0.0099 (14)
C28	0.0731 (18)	0.103 (2)	0.0617 (18)	0.0115 (17)	0.0265 (15)	−0.0069 (17)
C29	0.097 (2)	0.079 (2)	0.0426 (15)	0.0362 (17)	0.0181 (15)	0.0000 (14)
C30	0.093 (2)	0.0637 (18)	0.0516 (16)	0.0205 (16)	−0.0037 (15)	−0.0136 (13)
C31	0.0664 (15)	0.0543 (15)	0.0569 (16)	0.0101 (12)	0.0030 (13)	−0.0108 (12)
C32	0.0415 (10)	0.0367 (11)	0.0485 (13)	0.0046 (8)	0.0028 (9)	−0.0018 (9)
C33	0.0530 (13)	0.0498 (14)	0.0636 (16)	0.0129 (10)	0.0104 (12)	0.0042 (12)
C34	0.0661 (16)	0.0476 (15)	0.0799 (19)	0.0211 (12)	0.0066 (14)	0.0075 (13)
C35	0.0801 (18)	0.0388 (14)	0.0773 (19)	0.0054 (12)	0.0007 (15)	0.0135 (13)
C36	0.0589 (14)	0.0495 (15)	0.082 (2)	−0.0036 (12)	0.0088 (14)	0.0159 (14)
C37	0.0479 (12)	0.0418 (13)	0.0668 (16)	0.0022 (10)	0.0035 (11)	0.0058 (11)
C38	0.0324 (9)	0.0442 (12)	0.0457 (12)	0.0041 (8)	0.0067 (8)	−0.0028 (10)
C39	0.0470 (12)	0.0560 (15)	0.0589 (15)	−0.0035 (10)	0.0054 (11)	0.0036 (12)
C40	0.0485 (13)	0.0524 (15)	0.094 (2)	−0.0106 (11)	0.0154 (14)	−0.0067 (15)
C41	0.0369 (11)	0.0715 (18)	0.0768 (19)	−0.0007 (11)	0.0044 (12)	−0.0318 (15)
C42	0.0509 (13)	0.0739 (18)	0.0474 (14)	0.0114 (12)	0.0018 (11)	−0.0185 (13)
C43	0.0460 (11)	0.0516 (14)	0.0446 (13)	0.0057 (10)	0.0090 (10)	−0.0065 (10)
C44	0.0578 (13)	0.0340 (12)	0.0498 (14)	−0.0071 (9)	0.0075 (11)	−0.0050 (10)
C45	0.0475 (12)	0.0447 (13)	0.0523 (14)	−0.0011 (10)	−0.0024 (11)	−0.0052 (11)
C46	0.0439 (11)	0.0491 (14)	0.0567 (15)	−0.0063 (10)	0.0090 (11)	−0.0108 (11)
C47	0.0388 (10)	0.0403 (12)	0.0544 (14)	0.0031 (8)	0.0076 (10)	−0.0033 (11)
C48	0.0455 (12)	0.0411 (13)	0.0684 (16)	−0.0056 (9)	0.0015 (11)	−0.0060 (11)
C49	0.0447 (11)	0.0347 (12)	0.0652 (16)	−0.0002 (9)	0.0104 (11)	−0.0002 (11)
C50	0.0488 (12)	0.0423 (13)	0.0521 (14)	0.0028 (9)	0.0057 (11)	−0.0063 (11)
C51	0.0497 (12)	0.0349 (12)	0.0554 (14)	−0.0004 (9)	0.0029 (10)	−0.0004 (10)
C52	0.0491 (12)	0.0371 (12)	0.0548 (15)	0.0075 (9)	0.0013 (11)	0.0018 (10)

### Geometric parameters (Å, °)

**Table:**

Ru1—C45	1.883 (2)	C11—H11A	0.9300
Ru1—C44	1.922 (2)	C12—H12A	0.9300
Ru1—C46	1.926 (3)	C13—H13A	0.9700
Ru1—As1	2.4342 (3)	C13—H13B	0.9700
Ru1—Ru2	2.8507 (2)	C14—C15	1.376 (3)
Ru1—Ru3	2.8745 (2)	C14—C19	1.382 (3)
Ru2—C48	1.885 (2)	C15—C16	1.378 (4)
Ru2—C47	1.922 (2)	C15—H15A	0.9300
Ru2—C49	1.926 (3)	C16—C17	1.369 (4)
Ru2—As2	2.4374 (2)	C16—H16A	0.9300
Ru2—Ru3	2.8392 (2)	C17—C18	1.368 (4)
Ru3—C51	1.871 (2)	C17—H17A	0.9300
Ru3—C50	1.924 (3)	C18—C19	1.376 (3)
Ru3—C52	1.939 (3)	C18—H18A	0.9300
Ru3—P1	2.3334 (5)	C19—H19A	0.9300
As1—C7	1.931 (2)	C20—C21	1.370 (3)
As1—C1	1.950 (2)	C20—C25	1.376 (4)
As1—C13	1.9589 (19)	C21—C22	1.376 (5)
As2—C20	1.944 (2)	C21—H21A	0.9300
As2—C14	1.949 (2)	C22—C23	1.359 (7)
As2—C13	1.9668 (19)	C22—H22A	0.9300
P1—C38	1.820 (2)	C23—C24	1.367 (6)
P1—C26	1.836 (2)	C23—H23A	0.9300
P1—C32	1.838 (2)	C24—C25	1.393 (4)
F1—C29	1.360 (3)	C24—H24A	0.9300
F2—C35	1.354 (3)	C25—H25A	0.9300
F3—C41	1.360 (3)	C26—C27	1.382 (3)
O1—C44	1.145 (3)	C26—C31	1.389 (3)
O2—C45	1.138 (3)	C27—C28	1.388 (4)
O3—C46	1.145 (3)	C27—H27A	0.9300
O4—C47	1.142 (3)	C28—C29	1.353 (4)
O5—C48	1.138 (3)	C28—H28A	0.9300
O6—C49	1.141 (3)	C29—C30	1.356 (4)
O7—C50	1.152 (3)	C30—C31	1.388 (4)
O8—C51	1.144 (3)	C30—H30A	0.9300
O9—C52	1.138 (3)	C31—H31A	0.9300
C1—C6	1.373 (3)	C32—C37	1.381 (3)
C1—C2	1.374 (3)	C32—C33	1.396 (3)
C2—C3	1.386 (4)	C33—C34	1.383 (3)
C2—H2A	0.9300	C33—H33A	0.9300
C3—C4	1.364 (4)	C34—C35	1.367 (4)
C3—H3A	0.9300	C34—H34A	0.9300
C4—C5	1.357 (4)	C35—C36	1.358 (4)
C4—H4A	0.9300	C36—C37	1.388 (3)
C5—C6	1.386 (3)	C36—H36A	0.9300
C5—H5A	0.9300	C37—H37A	0.9300
C6—H6A	0.9300	C38—C43	1.387 (3)
C7—C8	1.369 (3)	C38—C39	1.395 (3)
C7—C12	1.387 (3)	C39—C40	1.384 (3)
C8—C9	1.382 (4)	C39—H39A	0.9300
C8—H8A	0.9300	C40—C41	1.369 (4)
C9—C10	1.371 (4)	C40—H40A	0.9300
C9—H9A	0.9300	C41—C42	1.349 (4)
C10—C11	1.358 (4)	C42—C43	1.383 (3)
C10—H10A	0.9300	C42—H42A	0.9300
C11—C12	1.379 (3)	C43—H43A	0.9300
			
C45—Ru1—C44	91.54 (10)	As1—C13—H13B	108.9
C45—Ru1—C46	91.41 (10)	As2—C13—H13B	108.9
C44—Ru1—C46	175.72 (9)	H13A—C13—H13B	107.7
C45—Ru1—As1	100.14 (7)	C15—C14—C19	118.5 (2)
C44—Ru1—As1	89.90 (7)	C15—C14—As2	118.58 (17)
C46—Ru1—As1	92.63 (7)	C19—C14—As2	122.92 (17)
C45—Ru1—Ru2	166.13 (7)	C14—C15—C16	120.6 (3)
C44—Ru1—Ru2	81.39 (7)	C14—C15—H15A	119.7
C46—Ru1—Ru2	95.07 (7)	C16—C15—H15A	119.7
As1—Ru1—Ru2	91.810 (7)	C17—C16—C15	120.4 (3)
C45—Ru1—Ru3	110.63 (7)	C17—C16—H16A	119.8
C44—Ru1—Ru3	99.93 (6)	C15—C16—H16A	119.8
C46—Ru1—Ru3	76.12 (7)	C18—C17—C16	119.5 (3)
As1—Ru1—Ru3	147.251 (9)	C18—C17—H17A	120.3
Ru2—Ru1—Ru3	59.460 (5)	C16—C17—H17A	120.3
C48—Ru2—C47	93.55 (10)	C17—C18—C19	120.3 (3)
C48—Ru2—C49	89.56 (10)	C17—C18—H18A	119.8
C47—Ru2—C49	175.70 (9)	C19—C18—H18A	119.8
C48—Ru2—As2	102.41 (7)	C18—C19—C14	120.6 (2)
C47—Ru2—As2	88.86 (6)	C18—C19—H19A	119.7
C49—Ru2—As2	93.37 (6)	C14—C19—H19A	119.7
C48—Ru2—Ru3	100.99 (7)	C21—C20—C25	119.2 (2)
C47—Ru2—Ru3	89.69 (6)	C21—C20—As2	121.3 (2)
C49—Ru2—Ru3	86.80 (6)	C25—C20—As2	119.48 (19)
As2—Ru2—Ru3	156.606 (9)	C20—C21—C22	120.0 (4)
C48—Ru2—Ru1	161.07 (7)	C20—C21—H21A	120.0
C47—Ru2—Ru1	82.33 (6)	C22—C21—H21A	120.0
C49—Ru2—Ru1	93.77 (7)	C23—C22—C21	120.6 (4)
As2—Ru2—Ru1	95.997 (7)	C23—C22—H22A	119.7
Ru3—Ru2—Ru1	60.687 (6)	C21—C22—H22A	119.7
C51—Ru3—C50	100.53 (10)	C22—C23—C24	120.6 (3)
C51—Ru3—C52	93.07 (10)	C22—C23—H23A	119.7
C50—Ru3—C52	166.40 (10)	C24—C23—H23A	119.7
C51—Ru3—P1	98.01 (7)	C23—C24—C25	118.7 (4)
C50—Ru3—P1	87.86 (7)	C23—C24—H24A	120.6
C52—Ru3—P1	90.09 (6)	C25—C24—H24A	120.6
C51—Ru3—Ru2	86.13 (7)	C20—C25—C24	120.8 (3)
C50—Ru3—Ru2	91.60 (7)	C20—C25—H25A	119.6
C52—Ru3—Ru2	89.48 (6)	C24—C25—H25A	119.6
P1—Ru3—Ru2	175.856 (15)	C27—C26—C31	118.3 (2)
C51—Ru3—Ru1	143.71 (7)	C27—C26—P1	124.42 (19)
C50—Ru3—Ru1	70.56 (7)	C31—C26—P1	117.28 (18)
C52—Ru3—Ru1	98.46 (6)	C26—C27—C28	121.2 (3)
P1—Ru3—Ru1	116.158 (15)	C26—C27—H27A	119.4
Ru2—Ru3—Ru1	59.854 (5)	C28—C27—H27A	119.4
C7—As1—C1	99.13 (9)	C29—C28—C27	118.3 (3)
C7—As1—C13	105.49 (9)	C29—C28—H28A	120.9
C1—As1—C13	100.42 (8)	C27—C28—H28A	120.9
C7—As1—Ru1	117.21 (6)	C28—C29—C30	123.1 (3)
C1—As1—Ru1	117.69 (7)	C28—C29—F1	118.6 (3)
C13—As1—Ru1	114.45 (6)	C30—C29—F1	118.3 (3)
C20—As2—C14	103.52 (10)	C29—C30—C31	118.5 (3)
C20—As2—C13	100.82 (9)	C29—C30—H30A	120.8
C14—As2—C13	104.04 (9)	C31—C30—H30A	120.8
C20—As2—Ru2	116.82 (6)	C30—C31—C26	120.7 (3)
C14—As2—Ru2	115.38 (6)	C30—C31—H31A	119.6
C13—As2—Ru2	114.36 (6)	C26—C31—H31A	119.6
C38—P1—C26	104.67 (10)	C37—C32—C33	118.2 (2)
C38—P1—C32	103.13 (10)	C37—C32—P1	121.90 (16)
C26—P1—C32	100.23 (10)	C33—C32—P1	119.87 (17)
C38—P1—Ru3	113.52 (7)	C34—C33—C32	120.8 (2)
C26—P1—Ru3	116.15 (7)	C34—C33—H33A	119.6
C32—P1—Ru3	117.26 (7)	C32—C33—H33A	119.6
C6—C1—C2	118.6 (2)	C35—C34—C33	118.5 (2)
C6—C1—As1	119.56 (16)	C35—C34—H34A	120.7
C2—C1—As1	121.83 (18)	C33—C34—H34A	120.7
C1—C2—C3	120.3 (3)	F2—C35—C36	119.0 (3)
C1—C2—H2A	119.8	F2—C35—C34	118.3 (2)
C3—C2—H2A	119.8	C36—C35—C34	122.7 (2)
C4—C3—C2	120.3 (3)	C35—C36—C37	118.4 (2)
C4—C3—H3A	119.8	C35—C36—H36A	120.8
C2—C3—H3A	119.8	C37—C36—H36A	120.8
C5—C4—C3	119.9 (3)	C32—C37—C36	121.3 (2)
C5—C4—H4A	120.1	C32—C37—H37A	119.4
C3—C4—H4A	120.1	C36—C37—H37A	119.4
C4—C5—C6	120.1 (3)	C43—C38—C39	118.2 (2)
C4—C5—H5A	119.9	C43—C38—P1	121.32 (17)
C6—C5—H5A	119.9	C39—C38—P1	120.17 (17)
C1—C6—C5	120.7 (2)	C40—C39—C38	121.2 (2)
C1—C6—H6A	119.6	C40—C39—H39A	119.4
C5—C6—H6A	119.6	C38—C39—H39A	119.4
C8—C7—C12	118.8 (2)	C41—C40—C39	117.7 (2)
C8—C7—As1	124.98 (16)	C41—C40—H40A	121.1
C12—C7—As1	116.15 (17)	C39—C40—H40A	121.1
C7—C8—C9	120.2 (2)	C42—C41—F3	118.7 (3)
C7—C8—H8A	119.9	C42—C41—C40	123.2 (2)
C9—C8—H8A	119.9	F3—C41—C40	118.1 (3)
C10—C9—C8	120.4 (3)	C41—C42—C43	119.0 (2)
C10—C9—H9A	119.8	C41—C42—H42A	120.5
C8—C9—H9A	119.8	C43—C42—H42A	120.5
C11—C10—C9	119.9 (3)	C42—C43—C38	120.7 (2)
C11—C10—H10A	120.0	C42—C43—H43A	119.7
C9—C10—H10A	120.0	C38—C43—H43A	119.7
C10—C11—C12	120.0 (3)	O1—C44—Ru1	173.5 (2)
C10—C11—H11A	120.0	O2—C45—Ru1	176.6 (2)
C12—C11—H11A	120.0	O3—C46—Ru1	170.8 (2)
C11—C12—C7	120.6 (2)	O4—C47—Ru2	174.32 (19)
C11—C12—H12A	119.7	O5—C48—Ru2	178.3 (2)
C7—C12—H12A	119.7	O6—C49—Ru2	174.7 (2)
As1—C13—As2	113.22 (9)	O7—C50—Ru3	169.1 (2)
As1—C13—H13A	108.9	O8—C51—Ru3	177.4 (2)
As2—C13—H13A	108.9	O9—C52—Ru3	174.4 (2)
			
C45—Ru1—Ru2—C48	62.5 (4)	C7—As1—C1—C2	−22.1 (2)
C44—Ru1—Ru2—C48	122.5 (3)	C13—As1—C1—C2	85.6 (2)
C46—Ru1—Ru2—C48	−55.1 (3)	Ru1—As1—C1—C2	−149.55 (18)
As1—Ru1—Ru2—C48	−147.9 (2)	C6—C1—C2—C3	−1.1 (4)
Ru3—Ru1—Ru2—C48	15.5 (2)	As1—C1—C2—C3	178.6 (2)
C45—Ru1—Ru2—C47	140.9 (3)	C1—C2—C3—C4	1.3 (5)
C44—Ru1—Ru2—C47	−159.06 (9)	C2—C3—C4—C5	−0.7 (5)
C46—Ru1—Ru2—C47	23.38 (10)	C3—C4—C5—C6	−0.1 (5)
As1—Ru1—Ru2—C47	−69.43 (6)	C2—C1—C6—C5	0.3 (4)
Ru3—Ru1—Ru2—C47	93.98 (6)	As1—C1—C6—C5	−179.4 (2)
C45—Ru1—Ru2—C49	−37.3 (3)	C4—C5—C6—C1	0.3 (4)
C44—Ru1—Ru2—C49	22.77 (9)	C1—As1—C7—C8	110.6 (2)
C46—Ru1—Ru2—C49	−154.80 (10)	C13—As1—C7—C8	7.0 (2)
As1—Ru1—Ru2—C49	112.40 (6)	Ru1—As1—C7—C8	−121.69 (19)
Ru3—Ru1—Ru2—C49	−84.19 (6)	C1—As1—C7—C12	−65.70 (19)
C45—Ru1—Ru2—As2	−131.0 (3)	C13—As1—C7—C12	−169.26 (18)
C44—Ru1—Ru2—As2	−71.02 (7)	Ru1—As1—C7—C12	62.03 (19)
C46—Ru1—Ru2—As2	111.41 (7)	C12—C7—C8—C9	1.7 (4)
As1—Ru1—Ru2—As2	18.605 (9)	As1—C7—C8—C9	−174.5 (2)
Ru3—Ru1—Ru2—As2	−177.986 (9)	C7—C8—C9—C10	−0.2 (5)
C45—Ru1—Ru2—Ru3	46.9 (3)	C8—C9—C10—C11	−1.4 (5)
C44—Ru1—Ru2—Ru3	106.96 (7)	C9—C10—C11—C12	1.4 (5)
C46—Ru1—Ru2—Ru3	−70.61 (7)	C10—C11—C12—C7	0.1 (5)
As1—Ru1—Ru2—Ru3	−163.409 (9)	C8—C7—C12—C11	−1.7 (4)
C48—Ru2—Ru3—C51	18.29 (11)	As1—C7—C12—C11	174.8 (2)
C47—Ru2—Ru3—C51	111.85 (10)	C7—As1—C13—As2	−96.54 (12)
C49—Ru2—Ru3—C51	−70.64 (10)	C1—As1—C13—As2	160.85 (11)
As2—Ru2—Ru3—C51	−161.73 (8)	Ru1—As1—C13—As2	33.79 (13)
Ru1—Ru2—Ru3—C51	−166.78 (7)	C20—As2—C13—As1	−141.78 (12)
C48—Ru2—Ru3—C50	118.73 (11)	C14—As2—C13—As1	111.17 (11)
C47—Ru2—Ru3—C50	−147.71 (9)	Ru2—As2—C13—As1	−15.57 (13)
C49—Ru2—Ru3—C50	29.80 (10)	C20—As2—C14—C15	104.4 (2)
As2—Ru2—Ru3—C50	−61.29 (7)	C13—As2—C14—C15	−150.6 (2)
Ru1—Ru2—Ru3—C50	−66.34 (7)	Ru2—As2—C14—C15	−24.5 (2)
C48—Ru2—Ru3—C52	−74.83 (11)	C20—As2—C14—C19	−77.1 (2)
C47—Ru2—Ru3—C52	18.73 (9)	C13—As2—C14—C19	27.9 (2)
C49—Ru2—Ru3—C52	−163.76 (10)	Ru2—As2—C14—C19	154.01 (16)
As2—Ru2—Ru3—C52	105.15 (7)	C19—C14—C15—C16	−0.8 (4)
Ru1—Ru2—Ru3—C52	100.10 (7)	As2—C14—C15—C16	177.8 (3)
C48—Ru2—Ru3—Ru1	−174.93 (8)	C14—C15—C16—C17	−1.0 (5)
C47—Ru2—Ru3—Ru1	−81.37 (7)	C15—C16—C17—C18	1.6 (5)
C49—Ru2—Ru3—Ru1	96.14 (7)	C16—C17—C18—C19	−0.5 (5)
As2—Ru2—Ru3—Ru1	5.05 (2)	C17—C18—C19—C14	−1.3 (4)
C45—Ru1—Ru3—C51	−146.55 (15)	C15—C14—C19—C18	1.9 (4)
C44—Ru1—Ru3—C51	−51.09 (14)	As2—C14—C19—C18	−176.55 (19)
C46—Ru1—Ru3—C51	127.24 (14)	C14—As2—C20—C21	−19.8 (2)
As1—Ru1—Ru3—C51	54.51 (12)	C13—As2—C20—C21	−127.3 (2)
Ru2—Ru1—Ru3—C51	22.67 (12)	Ru2—As2—C20—C21	108.2 (2)
C45—Ru1—Ru3—C50	−65.37 (11)	C14—As2—C20—C25	162.3 (2)
C44—Ru1—Ru3—C50	30.08 (10)	C13—As2—C20—C25	54.8 (2)
C46—Ru1—Ru3—C50	−151.59 (10)	Ru2—As2—C20—C25	−69.7 (2)
As1—Ru1—Ru3—C50	135.69 (7)	C25—C20—C21—C22	−0.8 (4)
Ru2—Ru1—Ru3—C50	103.84 (7)	As2—C20—C21—C22	−178.7 (3)
C45—Ru1—Ru3—C52	106.35 (10)	C20—C21—C22—C23	0.4 (6)
C44—Ru1—Ru3—C52	−158.19 (10)	C21—C22—C23—C24	−0.2 (7)
C46—Ru1—Ru3—C52	20.14 (10)	C22—C23—C24—C25	0.4 (7)
As1—Ru1—Ru3—C52	−52.59 (7)	C21—C20—C25—C24	1.0 (4)
Ru2—Ru1—Ru3—C52	−84.43 (7)	As2—C20—C25—C24	179.0 (2)
C45—Ru1—Ru3—P1	12.06 (8)	C23—C24—C25—C20	−0.8 (5)
C44—Ru1—Ru3—P1	107.52 (7)	C38—P1—C26—C27	−3.9 (2)
C46—Ru1—Ru3—P1	−74.15 (7)	C32—P1—C26—C27	102.7 (2)
As1—Ru1—Ru3—P1	−146.88 (2)	Ru3—P1—C26—C27	−129.9 (2)
Ru2—Ru1—Ru3—P1	−178.723 (18)	C38—P1—C26—C31	178.21 (18)
C45—Ru1—Ru3—Ru2	−169.22 (8)	C32—P1—C26—C31	−75.17 (19)
C44—Ru1—Ru3—Ru2	−73.76 (7)	Ru3—P1—C26—C31	52.2 (2)
C46—Ru1—Ru3—Ru2	104.57 (7)	C31—C26—C27—C28	−1.5 (4)
As1—Ru1—Ru3—Ru2	31.841 (15)	P1—C26—C27—C28	−179.4 (2)
C45—Ru1—As1—C7	−94.36 (10)	C26—C27—C28—C29	1.1 (5)
C44—Ru1—As1—C7	174.09 (9)	C27—C28—C29—C30	−0.1 (5)
C46—Ru1—As1—C7	−2.46 (9)	C27—C28—C29—F1	179.8 (3)
Ru2—Ru1—As1—C7	92.71 (7)	C28—C29—C30—C31	−0.4 (5)
Ru3—Ru1—As1—C7	65.67 (7)	F1—C29—C30—C31	179.6 (2)
C45—Ru1—As1—C1	23.77 (10)	C29—C30—C31—C26	0.0 (4)
C44—Ru1—As1—C1	−67.78 (9)	C27—C26—C31—C30	0.9 (4)
C46—Ru1—As1—C1	115.67 (9)	P1—C26—C31—C30	179.0 (2)
Ru2—Ru1—As1—C1	−149.17 (7)	C38—P1—C32—C37	−114.8 (2)
Ru3—Ru1—As1—C1	−176.21 (7)	C26—P1—C32—C37	137.4 (2)
C45—Ru1—As1—C13	141.34 (10)	Ru3—P1—C32—C37	10.7 (2)
C44—Ru1—As1—C13	49.79 (10)	C38—P1—C32—C33	63.4 (2)
C46—Ru1—As1—C13	−126.76 (10)	C26—P1—C32—C33	−44.4 (2)
Ru2—Ru1—As1—C13	−31.59 (7)	Ru3—P1—C32—C33	−171.08 (16)
Ru3—Ru1—As1—C13	−58.63 (7)	C37—C32—C33—C34	0.0 (4)
C48—Ru2—As2—C20	−72.35 (12)	P1—C32—C33—C34	−178.2 (2)
C47—Ru2—As2—C20	−165.73 (11)	C32—C33—C34—C35	0.4 (4)
C49—Ru2—As2—C20	17.94 (11)	C33—C34—C35—F2	179.5 (3)
Ru3—Ru2—As2—C20	107.67 (9)	C33—C34—C35—C36	−0.6 (5)
Ru1—Ru2—As2—C20	112.10 (9)	F2—C35—C36—C37	−179.7 (3)
C48—Ru2—As2—C14	49.64 (11)	C34—C35—C36—C37	0.4 (4)
C47—Ru2—As2—C14	−43.74 (9)	C33—C32—C37—C36	−0.2 (4)
C49—Ru2—As2—C14	139.93 (10)	P1—C32—C37—C36	178.0 (2)
Ru3—Ru2—As2—C14	−130.34 (7)	C35—C36—C37—C32	0.0 (4)
Ru1—Ru2—As2—C14	−125.91 (7)	C26—P1—C38—C43	127.61 (17)
C48—Ru2—As2—C13	170.28 (11)	C32—P1—C38—C43	23.16 (19)
C47—Ru2—As2—C13	76.90 (10)	Ru3—P1—C38—C43	−104.75 (16)
C49—Ru2—As2—C13	−99.43 (10)	C26—P1—C38—C39	−59.03 (19)
Ru3—Ru2—As2—C13	−9.70 (8)	C32—P1—C38—C39	−163.47 (17)
Ru1—Ru2—As2—C13	−5.27 (7)	Ru3—P1—C38—C39	68.62 (18)
C51—Ru3—P1—C38	162.57 (11)	C43—C38—C39—C40	−1.8 (3)
C50—Ru3—P1—C38	62.24 (10)	P1—C38—C39—C40	−175.41 (18)
C52—Ru3—P1—C38	−104.31 (10)	C38—C39—C40—C41	1.0 (4)
Ru1—Ru3—P1—C38	−4.83 (8)	C39—C40—C41—C42	0.6 (4)
C51—Ru3—P1—C26	−76.00 (11)	C39—C40—C41—F3	178.7 (2)
C50—Ru3—P1—C26	−176.34 (11)	F3—C41—C42—C43	−179.3 (2)
C52—Ru3—P1—C26	17.11 (11)	C40—C41—C42—C43	−1.2 (4)
Ru1—Ru3—P1—C26	116.59 (9)	C41—C42—C43—C38	0.3 (3)
C51—Ru3—P1—C32	42.38 (11)	C39—C38—C43—C42	1.2 (3)
C50—Ru3—P1—C32	−57.95 (10)	P1—C38—C43—C42	174.69 (16)
C52—Ru3—P1—C32	135.50 (10)	C51—Ru3—C50—O7	−60.1 (11)
Ru1—Ru3—P1—C32	−125.03 (8)	C52—Ru3—C50—O7	119.1 (11)
C7—As1—C1—C6	157.51 (19)	P1—Ru3—C50—O7	37.6 (11)
C13—As1—C1—C6	−94.8 (2)	Ru2—Ru3—C50—O7	−146.5 (11)
Ru1—As1—C1—C6	30.1 (2)	Ru1—Ru3—C50—O7	156.4 (11)

### Hydrogen-bond geometry (Å, °)

**Table:**

*D*—H···*A*	*D*—H	H···*A*	*D*···*A*	*D*—H···*A*
C23—H23A···F3^i^	0.93	2.46	3.337 (5)	157
C34—H34A···O3^ii^	0.93	2.58	3.405 (3)	148
C42—H42A···O2^iii^	0.93	2.52	3.438 (3)	167
C11—H11A···Cg1^iv^	0.93	2.94	3.767 (3)	149
C28—H28A···Cg2^ii^	0.93	2.89	3.807 (3)	168

Symmetry codes: (i) *x*+1, *y*, *z*; (ii) −*x*+1, *y*+1/2, −*z*+1/2; (iii) −*x*+1, −*y*, −*z*; (iv) −*x*+1, *y*−1/2, −*z*+1/2.
